# Participation in a free medication program among non-registered permanent residents with severe mental disorders in Bao’an district, Shenzhen, China and its influencing factors

**DOI:** 10.1186/s12888-021-03485-8

**Published:** 2021-09-30

**Authors:** Chu-Hong Lu, Jian-Hu Zhong, Ying-Dong Lv, Jia-Li Luo, Juan Cheng, Li Qing, Qing Chen, Cheng-feng Liu

**Affiliations:** 1 Shenzhen Bao’an Center for Chronic Disease Control, Shenzhen, China; 2 Songgang People’ Hospital, Bao’an District, Shenzhen, China; 3 Affiliated Cancer Hospital and Institute of Guangzhou Medical University, Guangzhou, China

**Keywords:** Free medication program, Non-registered permanent residents, Severe mental disorders

## Abstract

**Background:**

In 2016, the government of Bao’an District, Shenzhen, China launched a free medication program for all non-registered permanent residents with severe mental disorders (SMD) within its jurisdiction, in efforts to reduce the relapse caused by intermittent medication or non-medication. Participation in the program has not been analyzed since its inauguration. This study aimed to evaluate the participation of non-registered permanent residents with SMD in the program from 2016 to 2020 and to explore its influencing factors.

**Methods:**

This is a retrospective cross-sectional study of 3760 non-registered permanent residents with SMD in Bao’an District, Shenzhen, China (response rate: 78.64%). Data have been obtained from two sources: the Shenzhen Information System for Psychosis in 2020 and the free medication program’s management files from 2016 to 2020. We employ descriptive statistics to analyze the participation rate of the free medication program among non-registered permanent patients. Logistic regression analysis is used to explore the factors affecting the patients’ participation in the program.

**Results:**

The participation rate of the free medication program among non-registered permanent patients has shown an upward trend, rising from 28.83% in 2016 to 58.32% in 2020. High participation rates have been registered among the following patient subgroups: those aged between 30 and 39 (63.11%), those with high school/technical secondary school (62.33%), those from rural areas (61.62%), those living in poverty (67.79%), those suffering schizoaffective disorder (72.26%), those having SMD for less than 5 years (59.89%), and those with family history of mental illness (71.23%).Logistics regression analysis shows that age, patient-guardian relationship, place of residence, financial condition, types of disease, duration of illness and family history are the main factors affecting the patients’ decision to participate in the free medication program.

**Conclusion:**

The steadily increasing participation rate of the free medication program indicates that the program has been gradually accepted and recognized by non-registered permanent residents with SMD. However, nearly half of the patients have yet to join the program. To further raise the participation rate, special attention should be given to patients who are financially secure, aged below 20, without guardians, intellectually disabled or suffering SMD for over 5 years.

## Background

According to the Mental Health Law of the People’s Republic of China, severe mental disorders (SMD) refer to mental illnesses with serious symptoms that damage the patients’ social adaption and other functions and cause their failure to fully understand the reality, realize their own health condition or deal with their personal affairs [1]. SMD include schizophrenia, schizoaffective disorder, delusional disorder, bipolar disorder, psychotic disorder due to epilepsy and intellectual disability with mental disorders. SMD are also found in patients who have conducted behaviors threatening others’ safety and those diagnosed with the risk of endangering others [1]. By the end of 2017, China had registered 5.81 million people with SMD [2]. SMD are generally characterized by long course of illness, low cure rate and high recurrence rate. Due to the lack of long-term systematic and standardized treatment, many patients relapse quickly after being treated in the acute stage. Some patients may cause accidents under the influence of SMD with their social functions severely impaired by the illness, which brings a heavy burden to their families and society [3]. Medication is an essential part of the treatment for SMD patients, both in the acute stage and long-term treatment [4, 5]. According to studies of the medical needs of SMD patients, their most common need is financial aid, including free treatment and medical reimbursement, etc. [6]. In a bid to reduce the recurrence risk for patients due to intermittent medication or non-medication, local health foundations in China, subsidized by the central government, officially launched the treatment project for SMD patients (Project 686) in 2004. Demonstration areas have been established in provincial-level regions across the country to register and evaluate SMD patients, mainly those with schizophrenia. Doctors with township-level health centers and community health centers follow up the patients regularly, check their mental and physical condition, conduct risk assessment, and provid free medicine and hospital treatment for impoverished SMD patients [7]. Since then, local authorities across China have launched free medication programs for registered permanent residents with SMD [8–13]. Previous studies indicated that the implementation of free medication programs can significantly improve the mental condition, social functions, medication adherence and reemployment rate of SMD patients [14–18]. Free medication is of great significance to maintaining social stability and building a harmonious society [19].

Shenzhen is a coastal city in southern China covering a land area of 1997.47sq. km [20]. As a special economic zone, Shenzhen registered a GDP of 2.69 trillion RMB in 2019, ranked third among all cities in the Chinese mainland [20]. The megacity has a population of 13.44 million, including 8.49 million non-registered permanent residents, which account for 63.18% of the total and are out of proportion to the population of registered permanent residents [20]. Located in the west of Shenzhen, Bao’an District is the largest and most populated among the city’s 10 districts [20]. Bao’an has a developed economy and was ranked eighth on the list of China’s Top 100 Districts in 2018 [21]. The district has 10 sub-district offices under its jurisdiction and 2.69 million non-registered permanent residents, which make up 80.53% of the district’s total population [20]. Non-registered permanent residents paly a vital role in the economic development of China’s urban areas [22]. However, most social welfare programs in the urban areas are only available to registered permanent residents, but not to non-registered permanent residents [23–26]. It is the same situation with the free medication programs for SMD patients. In 2006, Bao’an District launched a free medication program for registered permanent residents with SMD. It was not until 2016 that the district introduced a free medication program for non-registered permanent residents with the illness, offering reduction of medication fees (300 RMB/person/month) and examination fees (800 RMB/person/year). The participation rate of the free medication program among non-registered permanent residents has not been evaluated since it was implemented. Previous relevant studies focused on comparing the situations before and after the implementation of free medication programs, but little academic attention has been paid to analyzing the participation of the programs and characteristics of the participants, especially non-registered permanent residents. This study aims to analyze the participation rate of the free medication program and its impacting factors so as to provide key research materials for improving management of and services for non-registered permanent residents with SMD in China’s developed areas.

## Methods

### Data sources

This is a retrospective cross-sectional study. Data used in this study have been obtained from two sources: the Shenzhen Information System for Psychosis in 2020 (database of Bao’an District) and the district’s management files of the free medication program from 2016 to 2020, both of which are managed by the professionals with the Bao’an Center for Chronic Disease Control. The professionals conduct regular checks and quality control management of the above two sources to to ensure the data’s authenticity, reliability, and accuracy. Data obtained from the Shenzhen Information System for Psychosis include (1) demographic information such as age, gender, education level, marital status, guardian, place of residence and financial condition; (2) clinical information such as type of disease, duration of illness and family history of mental illness. Information of the participants of Bao’an District’s free medication program, including their ID card number and date of application for the program, has been obtained from the program’s management files from 2016 to 2020.

### Participants

**Fig. 1:**
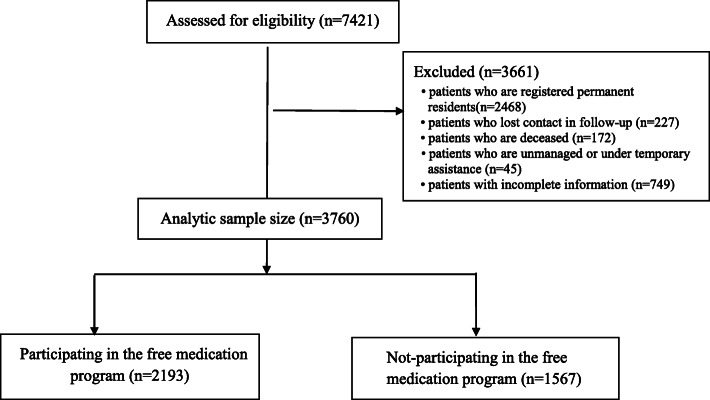
Flowchart illustrating the patient recruitment and exclusion processes in the study of the free medication program

Research subjects of this study are SMD patients in Bao’an District who have been diagnosed by medical institutions and registered in the Shenzhen Information System for Psychosis before December 31, 2020, meet the standard of non-registered permanent residents, and receive regular follow-up management by local communities. Patients who are deceased, losing contact in, unmanaged or under temporary assistance as well as 749 questionnaires with incomplete information have been excluded from this study. The subjects of the study include 3760 non-registered permanent residents with SMD. Figure 1, a flow diagram, describes how the subjects are recruited and excluded. All non-registered permanent residents with SMD, who are included in the Shenzhen Information System and receive regular follow-up management by local communities, can apply for the free medication program in Bao’an District. Doctors with the community health centers are required to inform all patients of the program during follow-up visits of the patients (face to face, by WeChat or phone calls, etc.) and receive clear replies from the patients about whether they will participate in the program. In addition, the doctors are required to assist the patients in application if they agree to participate. This study was approved by the Bao’an Center for Chronic Disease Control (Shenzhen, China). All methods were performed in accordance with the relevant guidelines and regulations.

### Description of related definitions

Non-registered permanent residents: according to China’s current demographic system and demographic monitoring methods issued by the Shenzhen municipal government, since 2006, non-registered permanent residents in Shenzhen refer to people who have lived in the city for over 6 months but do not have Hukou, or household registration status. Impoverished residents in Shenzhen refer to people living on government allowances whose monthly income is less than 1.5 times the minimum living standard in Shenzhen (1740 yuan). According to China’s National Basic Public Health Service Standard (third edition), guardian of SMD patients is a person who exercises the duties of guardianship according to law. Medication adherence is divided into three categories:regular medication, intermittent medication and non-medication. Regular medication means the patients take medicines according to doctor’s advice and does not stop, reduce, or increase the dosage of medicines by themselves. Intermittent medication means the patients do not follow doctor’s advice to take medicines or reduce the frequency and quantity of medication by themselves. Non-medication means that the patients do not take any of the medicines prescribed by doctors or that the doctors think the patients have recovered with no need for medication.

#### Statistical analysis

Statistical analysis is performed with SPSS 26.0. Descriptive statistics are used to analyze the quantity and ratio, and chi-square test is employed to compare the difference of demographic and clinical variables between different groups among non-registered permanent residents with SMD. Candidate variables with a *P* value < 0.2 on univariate analysis are included in multivariate logistic regression to evaluate factors associated with the participation rate of the free medication program among non-registered permanent residents with SMD. *P* values < 0.05 are considered statistically significant.

## Results

### Participation rate of free medication program in 2016–2020

By the end of 2020, there were 7421 SMD patients in Bao’an District, Shenzhen. Among them, 4953 were non-registered permanent residents, accounting for 66.74% of the total. Of the non-registered permanent residents with SMD, 172 were excluded from this study because they had deceased before the research started. After the recruitment and exclusion processes (Fig. 1), a total of 3760 individuals (with complete data) were recruited from the 4781 eligible non-registered permanent residents with SMD, and the overall response rate was 78.64%.Participation in the free medication program among non-registered permanent residents with SMD from 2016 to 2020 is presented in Table 1. From 2016 to 2020, the participation rate of the free medication program among non-registered permanent residents with SMD in Bao’an District, Shenzhen, is 28.83, 40.47, 43.73, 51.58 and 58.32%, respectively, and the participation rate shows an upward trend (Chi-square Trend =501.357, *P* < 0.001).

**Table 1:** Participation in the free medication program among non-registered permanent residents with SMD from 2016 to 2020

Year	Participating		Non-participating		Chi-square Trend	***P*** value
	N	%	N	%		
2016	517	28.83	1276	71.17	501.357	<0.001
2017	955	40.47	1405	59.53		
2018	1251	43.73	1610	56.27		
2019	1750	51.58	1643	48.42		
2020	2193	58.32	1567	41.68		

### Psychotropic drug and medication adherence of non-registered permanent residents with SMD

**Fig. 2:**
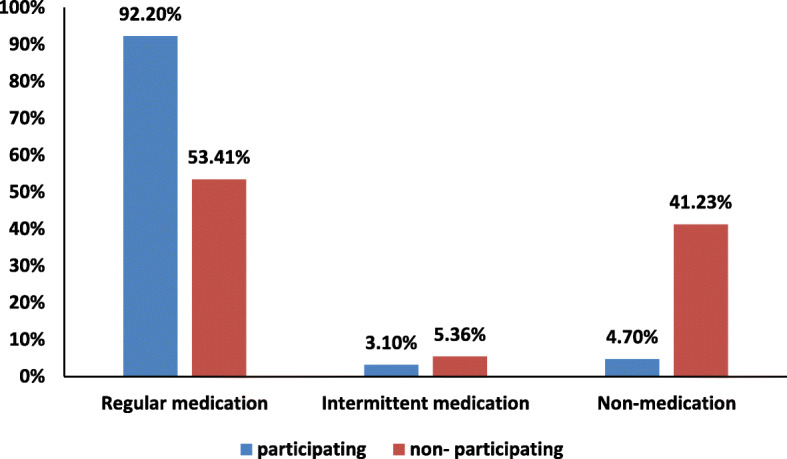
Medication adherence of non-registered permanent residents with SMD participating and not participating in free medication program

From 2016 to 2020, the cost of antipsychotic drugs for 2193 non-registered permanent residents with SMD in Bao’an District totaled 8.76 million RMB. The per capita cost was 3995.94 RMB. In 2020, olanzapine was the most frequently used antipsychotic drug among participants in the free medication program, followed by valproate, risperidone, aripiprazole, quetiapine, clozapine, amisulpride, lithium carbonate and benzodiazepines. Olanzapine was the most frequently used in intellectually disabled patients with mental disorders, followed by valproate, risperidone, amisulpride, aripiprazole, quetiapine, oxcarbazepine, clozapine and benzodiazepines. Figure 2 shows the medication adherence of patients participating in the free medication program and those not participating. 92.20% of the patients participating in the program are able to take regular medication, while only 53.41% of the non-participating patients take the drugs regularly. 3.10 and 4.70% of the patients participating in the program show intermittent medication and non-medication, respectively, compared with 5.36 and 41.23% in the non-participating patients.

### Demographic and clinical characteristics

The socio-demographic and clinical characteristics of the participating and non- participating patients are shown in Table 2. The study covers 3760 patients, of which 2193(58.32%) have participated in the free medication program and 1567 (41.68%) have not. The 30–39 age group registers the highest participation rate (63.11%, *P*<0.001), while the under-20 age group posts the lowest participation rate (30.15%, *P*<0.001). In terms of education level, patients with junior high school, and high school or technical secondary school diplomas show relatively high participation rates (62.24 and 62.33%, respectively), while illiterate or semiliterate patients record the lowest participation rate (39.38%, *P*<0.001). Married and divorced patients register relatively high participation rates (60.51 and 61.15%, respectively), and widowed patients show the lowest participation rate (48.00%, *P*<0.05). Patients whose guardian is a spouse have the highest participation rate (62.74%, *P*<0.001). The participation rate of patients living in rural areas is higher than that of patients living in urban areas or foreign countries (61.62%, *P*<0.001). The impoverished patients show higher participation rate than the financially secure ones (67.79%, *P*<0.001). Patients with schizoaffective disorder have the highest participation rate (72.26%, *P*<0.001) among all types of mental illness. Patients suffering SMD for less than 5 years show the highest participation rate (59.89%, *P*<0.05) than those with other duration of illness. Patients with family history of mental illness record higher participation rate than those without family history (71.23%, *P*<0.05).

**Table 2:** Socio-demographic and clinical characteristics of the participating and non- participating patients

Variable name	N=	%	Participating (***n***=2193)		Non- participating (***n***=1567)		Chi-square	***P*** value
			N	%	N	%		
Gender							2.474	0.116
Male	1805	48.01	1029	57.01	776	42.99		
Female	1955	51.99	1164	59.54	791	40.46		
Age (Years)							98.605	< 0.001
<20	262	6.97	79	30.15	183	69.85		
20 to 29	834	22.18	505	60.55	329	39.45		
30 to 39	1171	31.14	739	63.11	432	36.89		
40 to 49	626	16.65	370	59.11	256	40.89		
50 to 59	501	13.32	290	57.88	211	42.12		
≥60	366	9.73	210	57.38	156	42.62		
Education level							63.821	< 0.001
Illiteracy/semiliterate	320	8.51	126	39.38	194	60.63		
Primary school	659	17.53	367	55.69	292	44.31		
Junior high school	1462	38.88	910	62.24	552	37.76		
High school/technical secondary school	738	19.63	460	62.33	278	37.67		
College degree and above	581	15.45	330	56.80	251	43.20		
Marital status							11.909	0.008
Married	2036	54.15	1232	60.51	804	39.49		
Unmarried	1517	40.35	841	55.44	676	44.56		
Divorced	157	4.18	96	61.15	61	38.85		
Widowed	50	1.33	24	48.00	26	52.00		
Guardian							50.817	< 0.001
Spouse	1205	32.05	756	62.74	449	37.26		
Parents	1218	32.39	702	57.64	516	42.36		
Children	481	12.79	290	60.29	191	39.71		
Relative	582	15.48	333	57.22	249	42.78		
Friends and others	121	3.22	59	48.76	62	51.24		
None	153	4.07	53	34.64	100	65.36		
Residence							43.344	< 0.001
Urban /Foreign	1048	27.79	522	49.81	526	50.19		
Rural	2712	72.21	1671	61.62	1041	38.38		
Financial condition							34.073	< 0.001
Non-poverty	3018	80.27	1690	56.00	1328	44.00		
Poverty	742	19.73	503	67.79	239	32.21		
Types of disease							154.521	< 0.001
Schizophrenia	2351	62.53	1459	62.06	892	37.94		
Schizoaffective disorder	137	3.64	99	72.26	38	27.74		
Paranoid disorder	34	0.90	17	50.00	17	50.00		
Bipolar disorder	740	19.68	414	55.95	326	44.05		
Epileptic mental disorder	79	2.10	51	64.56	28	35.44		
Intellectual disability with mental disorders	249	6.62	58	23.29	191	76.71		
Other mental disorders	170	4.52	95	55.88	75	44.12		
Duration of illness (year)							6.079	0.048
<5	1790	47.61	1072	59.89	718	40.11		
5 to 9	1241	33.01	689	55.52	552	44.48		
≥10	729	19.39	432	59.26	297	40.74		
Family history of mental illness							5.103	0.024
Yes	73	1.94	52	71.23	21	28.77		
No	3687	98.06	2141	58.07	1546	41.93		

### Factors influencing participation in free medication program

The factors affecting the participation in the free medication program are listed in Table 3. Logistic regression analysis shows that using the under-20 age group as a reference group, the 30–39 age group’s participation rate is 2.66 times that of the reference group (OR = 2.66, 95% CI = 1.81–3.90),and the under-20 age group has the lowest participation rate. However, the patients’ education level and marital status are not associated with their participation rate. Using patients without guardians as a reference group, the participation rate of the patients with their parents as guardians is 3.64 times that of the reference group (OR = 3.64, 95% CI = 2.49–5.32). The participation rate of patients living in rural areas is 1.51 times that of those residing in urban areas or foreign countries (OR = 1.51, 95% CI = 1.30–1.77). The participation rate of impoverished patients is 1.63 times that of financially secure ones (OR = 1.63, 95% CI = 1.36–1.95). Using patients with schizophrenia as a reference group, patients with schizoaffective disorder have higher participation rate (OR = 1.56, 95% CI = 1.06–2.31), while those with bipolar disorder and with intellectual disability show lower participation rates (OR = 0.80, 95% CI = 0.67–0.96; OR = 0.27, 95%CI = 0.18–0.41). Patients suffering SMD for 5 to 9 years have lower participation rate, only 73% of the participation rate of those suffering the illness for less than 5 years (OR = 0.73, 95% CI = 0.62–0.85). The participation rate of patients with family history of mental illness is 1.84 times that of those without family history (OR = 1.84, 95% CI = 1.08–3.14).

**Table 3:** Factors influencing participation in the free medication program by logistic regression analyses

Variable name	B	S.E.	Wald	OR	95% CI		***p***-value
Gender							
Female				1			
Male	-0.02	0.08	0.04	0.99	0.85	1.14	0.838
Age (years)							
<20				1	-	-	-
20 to 29	0.78	0.18	18.18	2.18	1.52	3.12	0.001
30 to 39	0.98	0.20	25.09	2.66	1.81	3.90	< 0.001
40 to 49	0.80	0.22	13.54	2.22	1.45	3.40	< 0.001
50 to 59	0.72	0.23	9.65	2.05	1.30	3.21	0.002
≥60	0.72	0.26	7.71	2.06	1.24	3.42	0.005
Educational level							
Illiteracy/semiliterate				1			
Primary school	0.09	0.17	0.27	1.09	0.79	1.51	0.601
Junior high school	0.22	0.17	1.77	1.25	0.90	1.74	0.183
High school or technical secondary school	0.34	0.18	3.49	1.40	0.98	1.99	0.062
College degree and above	0.13	0.19	0.48	1.14	0.79	1.65	0.490
Marital status							
Unmarried				1			
Married	-0.05	0.13	0.18	0.95	0.74	1.22	0.676
Divorced	0.12	0.19	0.37	1.12	0.77	1.64	0.546
Widowed	-0.38	0.33	1.31	0.69	0.36	1.31	0.252
Guardian							
None				1			
Friends and others	0.66	0.26	6.47	1.93	1.16	3.19	0.011
Relative	0.91	0.20	21.71	2.49	1.70	3.66	< 0.001
Parents	1.29	0.19	44.26	3.64	2.49	5.32	< 0.001
Children	1.29	0.23	31.04	3.63	2.31	5.71	< 0.001
Mate	1.17	0.20	36.38	3.23	2.21	4.73	< 0.001
Residence							
Urban / Foreign				1			
Rural	0.41	0.08	27.18	1.51	1.30	1.77	< 0.001
Financial condition							
Non-poverty				1			
Poverty	0.49	0.09	28.32	1.63	1.36	1.95	< 0.001
Types of disease							
Schizophrenia				1			
Schizoaffective disorder	0.45	0.20	4.95	1.56	1.06	2.31	0.026
Paranoid disorder	-0.50	0.35	1.97	0.61	0.304	1.22	0.160
Bipolar disorder	-0.22	0.09	5.88	0.80	0.67	0.96	0.015
Epileptic mental disorder	0.08	0.25	0.11	1.08	0.67	1.77	0.746
Intellectual disability with mental disorders	-1.30	0.21	39.20	0.27	0.18	0.41	< 0.001
Other mental disorders	-0.36	0.17	4.67	0.70	0.51	0.97	0.031
Duration of illness (year)							
<5				1			
5 to 9	-0.32	0.08	15.59	0.73	0.62	0.85	< 0.001
≥10	-0.18	0.10	3.48	0.83	0.69	1.01	0.062
Family history of mental illness							
No				1			
Yes	0.61	0.27	4.96	1.84	1.08	3.14	0.026

## Discussion

This study is the first research into the participation of the free medication program in Bao’an District, Shenzhen, among non-registered permanent residents with SMD since the program was launched in 2016. As far as we know, this is also the first study in China to focus on the participation of non-registered permanent residents with SMD in the free medication program. Compared to the participation rate of registered permanent residents with SMD in Bao’an District in 2020(34.74%, not listed in the table), the participation rate (58.32%) of non-registered permanent patients is higher, according to this study. Non-registered permanent patients usually face more financial challenges than registered permanent patients and are more inclined to obtain subsidies to relieve their family of economic burden [10].

This study shows that although Bao’an District, Shenzhen, has higher medicine subsidies than Dahongmen District,Beijing [9], the participation rate of non-registered permanent patients in Bao’an in 2017 (40.47%) was still lower than that of registered permanent patients in Beijing in the same year (49%) [10], and the participation rate of non-registered permanent patients in Bao’an in 2018 (43.73%) was lower than that of registered permanent patients in Dahongmen in the same year (67.82%).The participation rate of non-registered permanent residents with schizophrenia in Bao’an stood at 62.06% in 2020, lower than that of registered permanent residents with the same disease in remote communities in southwest China’s Yunnan Province in 2013 (70.35%) [13]. In 2019, Bao’an District covers an area of 396.61 sq. km and has a permanent population of 3.34 million. However, due to the shortage of mental health resources, only one hospital in the district offered psychiatric outpatient services and undertook the free medication program. In order to improve the patients’ medication rate and optimize the program, Bao’an established clinical psychology departments in 6 hospitals in 2019, and each department is staffed with full-time psychiatrists by the end of 2020. The participation rate of the free medication program has increased steadily year by year, indicating that the program has been gradually accepted and recognized by non-registered permanent residents with SMD. However, nearly half of the patients have yet to join the program. This is an interesting discovery that may be related to the patients’ stigma consciousness, deficient awareness of the importance of medication [10, 13, 27], lack of access to metal health resources and medical care, and complicated application process in Bao’an District [12, 28, 29] This aspect can be further investigated in future studies to find out why some non-registered permanent residents have not participated in the free medication program.

The influencing factors discussed in this study are more focused on patient-related factors, including gender, age, education level, marital status, relationship with guardian, place of residence, financial condition, types of disease, duration of illness and family history. This study shows that patients aged below 20 have the lowest participation rate, while those aged between 30 and 39 have the highest participation rate. This finding is inconsistent with the research result of the free medication program among registered permanent patients in Dahongmen District, Beijing, which shows that age has no influence on the participation rate [9]. It is also in contradiction with the result of the free medication program among registered permanent patients in remote communities in Yunnan Province, which shows a negative correlation between age and participation rate [13]. One possible explanation for the inconsistencies is that among the patients aged below 20 in Bao’an, 59.54% are intellectually disabled patients with metal disorders, and patients with this disease has the lowest participation rate (only 12.18%). Compared to patients without guardians, those who have their parents, children, spouses, relatives and friends as guardians are more inclined to participate in the free medication program, especially if their parents are their guardians. One possible reason is that parents pay more attention to patients and are more willing to take care of patients with all their heart [30]. Poverty is a factor affecting the medication adherence of SMD patients [30, 31]. This study shows that impoverished patients are more likely to participate in the free medication program, which is consistent with previous research on the free medication program for registered permanent patients [10, 30–32]. The free medication program can reduce economic pressures for families, so patients from financially challenged families are more likely to participate in the program [10]. The study of Wang Xun et al. shows that the medication adherence of rural patients is lower than that of urban patients after discharge from hospitals [32]. In contrast, this study shows that rural patients are more inclined to participate in the free medication program than urban and foreign patients, and patients with family history of mental illness are more inclined to participate. This may be related to the patients’ financial condition. In addition, this study shows that education level and marital status have no impact on the participation rate of the free medication program in Bao’an, which is in line with the research result of the free medication program among registered permanent patients in Dahongmen [9].

This study shows that among all types of mental illness, intellectually disabled patients with mental disorders have the lowest participation rate, only 27% of that of patients with schizophrenia. This finding is inconsistent with the research result of the free medication program among unemployed, impoverished registered permanent residents with metal illness in Shanghai, which shows that patients with schizoaffective disorder have the lowest participation rate [12]. The low participation rate among intellectually disabled patients with mental disorders may be related to the low medication rate for this disease [27, 33]. These patient were usually diagnosed with intellectual disability at a very young age, which caused their family to give up treatment [27]. The low participation rate of these patients may also be related to the low education level of their family members, which lead to the latter’s failure to fully perform their role as guardians [33].

The participation rate of patients suffering SMD for 5 to 9 years is lower than that of patients suffering the disease for less than 5 years. This may be because patients with chronic diseases usually lack confidence in medical treatment and suffer from symptoms such as indolence and withdrawal [34, 35].

### Limitations

The free medication program in Bao’an District, Shenzhen, China covers all non-registered permanent residents with SMD within the district. It only applies to non-registered permanent residents (registered permanent patients are not applicable), making it difficult to evaluate the influencing factors with rigorous designs such as randomized controlled or comparative studies. The subjects of this study are limited to non-registered permanent residents with SMD in Bao’an District (which make up nearly 70% of SMD patients in the district), thus affecting the extrapolation of the research results. The data used in this study only contain the patients’ demographic and clinical characteristics, so the influencing factors discussed in the study focus more on patient-related factors. Lack of investigation into the specific reasons for the patients’ non-participation, including personal/family income and accessibility of medical care, makes it difficult to conduct further analysis. Moreover, although non-response bias is inevitable in this study, we believe non-response bias does not have large potential influence on our results, as characteristics of the excluded patients are similar to those participating in the study.

## Conclusions

This study showcases that the participation rate of the free medication program among non-registered permanent residents with SMD in Bao’an District, Shenzhen, has increased steadily year by year, from 28.83% in 2016 to 58.32% in 2020. The rising trend indicates that the program has been gradually accepted and recognized by the patients. However, nearly half of the patients have yet to agree to join the program. Further efforts should be made to improve the participation rate of patients who are financially secure, aged below 20, without guardians, suffering intellectually disability with mental disorder, or suffering SMD for over 5 years. Impoverished patients are more inclined to participate in the free medication program, suggesting that the program can indeed help poor families obtain psychiatric medications. According to these findings, this study recommends the following: (1) Efforts should be made to further understand the medication needs of non-registered permanent residents with SMD and the side effects of antipsychotic drugs so as to optimize the free medication program. (2) Doctors with community health centers should regularly follow up the patients and hold lectures on medication to improve the patients’ and their families’ understanding of medication adherence and the free medication programs.

## Data Availability

The datasets used and/or analysed during the current study are available from the corresponding author on reasonable request.

## Abbreviation

SMD: Severe mental disorders
